# Choice between decision-making strategies in human route-following

**DOI:** 10.3758/s13421-023-01422-6

**Published:** 2023-04-26

**Authors:** Otmar Bock, Ju-Yi Huang, Özgür A. Onur, Daniel Memmert

**Affiliations:** 1 Institute of Exercise Training and Sport Informatics, German Sport University Cologne, 50927 Cologne, Germany; 2 Department of Neurology, Faculty of Medicine and University Hospital Cologne, University of Cologne, Cologne, Germany

**Keywords:** Spatial navigation, Wayfinding, Serial order strategy, Associative cue strategy, Dual encoding

## Abstract

To follow a prescribed route, we must decide which way to turn at intersections. To do so, we can memorize either the serial order of directions or the associations between spatial cues and directions (“at the drug store, turn left”). Here, we investigate which of these two strategies is used if both are available. In Task S, all intersections looked exactly alike, and participants therefore had to use the serial order strategy to decide which way their route continued. In Task SA, each intersection displayed a unique spatial cue, and participants therefore could use either strategy. In Task A, each intersection displayed a unique cue, but the serial order of cues varied between trips, and participants therefore had to use the associative cue strategy. We found that route-following accuracy increased from trip to trip, was higher on routes with 12 rather than 18 intersections, and was higher on Task SA than on the other two tasks, both with 12 and with 18 intersections. Furthermore, participants on Task SA acquired substantial knowledge about the serial order of directions as well as about cue–direction associations, both with 12 and with 18 intersections. From this we conclude that, when both strategies were available, participants did not pick the better one but rather used both. This represents dual encoding, a phenomenon previously described for more elementary memory tasks. We further conclude that dual encoding may be implemented even if the memory load is not very high (i.e., even with only 12 intersections).

## Introduction

Finding our way in a city or building requires a complex interaction between sensory, cognitive, and motor processes (for a review, see, e.g., Hegarty et al., 2022; Wolbers & Hegarty, 2010). Those processes contribute to wayfinding in a flexible fashion, depending, for example, on the environmental topography, the availability of spatial cues such as conspicuous buildings or direction signs, the traveler’s prior knowledge, individual preferences, and the nature of the wayfinding task (Ekstrom et al., 2018; Hölscher et al., 2009; Wiener et al., 2009). Our study deals with one particular wayfinding task—namely, following a prescribed route. As an example, consider a person who was guided by a colleague from the hotel to the conference center on the first day of a meeting, and who endeavors to walk alone on the second day. The person remembers to turn left when leaving the hotel, and then to walk straight across two intersections. A drug store at the third intersection reminds the person to turn left, until the conference center is reached.

The above example illustrates that route-following is essentially a locomotion-decision cycle: Travelers walk towards an intersection, decide in which direction to continue, walk in that direction towards the next intersection, decide again, until they arrive at their destination. The above example also shows that route-following can be based on two distinct decision-making strategies. With the *serial order strategy*, travelers memorize a sequence of directions (Iglói et al., 2009; Tlauka & Wilson, 1994), such as “left, then straight, then straight again.” With the *associative cue strategy*, they memorize distinctive objects along the way and pair them with the direction to take (Tlauka & Wilson, 1994; Waller & Lippa, 2007), such as “turn left at the drug store.” The distinctive objects for the latter strategy are often called “spatial cues” or “landmarks.” They are not necessarily visual: Sounds and smells can also serve as spatial cues (Hamburger, 2020). Other decision-making strategies are available if the task is not to follow a prescribed route, but those strategies are beyond the scope of the present work.

Travelers who take repeated trips along a prescribed route will encounter the same spatial cues in the same order on each trip, and therefore they principally have the option to use the serial order strategy, the associative cue strategy, or a combination of both. It is well established that the choice of a given strategy depends on factors such as as environmental topography, sex, and individual preferences (Boone et al., 2018; Hölscher et al., 2009; Iaria et al., 2003), and that travelers can change their decision strategy between trips, or even in the middle of a given trip (Hamburger, 2020; Wolbers & Hegarty, 2010). To investigate route-following by a given strategy, therefore, authors have implemented wayfinding tasks that allow the use of only one particiular strategy. Thus, route-following by the serial order strategy alone has been investigated in a virtual maze whose corridors and intersections all looked exactly the same; since no distinctive objects were available, participants could not follow the route by recalling object-direction associations, but rather had to recall the serial order of directions (e.g., Jansen-Osmann, 2002; Lingwood et al., 2015; Tlauka & Wilson, 1994; Waller & Lippa, 2007). Similarly, route-following by the associative cue strategy alone has been investigated by providing a unique distinctive object at each maze intersection but varying the serial order of those objects from trip to trip (Bock & Borisova, 2022). For example, a particular tree might be encountered at the fifth intersection of the first trip, at the eighth intercection of the second trip, and so on, but it is always associated with the same direction on all trips. Participants therefore could not follow the route by recalling the serial order of directions but rather had to recall object-direction associations. We will refer to paradigms that call for the serial order strategy as “Task S,” to paradigms that call for the associative cue strategy as “Task A,” and to control paradigms that allow both strategies as “Task SA.” Note that only Task SA corresponds to our everyday experience: We normally encounter neither a series of intersections which look exactly the same, as on Task S, nor visual cues whose serial order varies from trip to trip, as on Task A. These deviations from everyday life are unavoidable in order to obtain strategy-specific tasks.

**Fig. 1:**
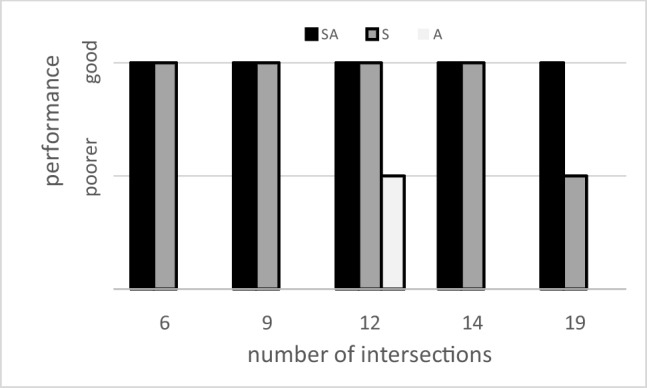
Summary of literature on route-following with Tasks SA, S, and A. *Note.* Performance is classified as “good” if it is not significantly different from Task SA, and as “poorer” if it is significantly lower than on Task SA. Each study used a fixed number of intersections, and that number differed between studies: either six intersections (Lingwood et al., 2015), or nine (Jansen-Osmann, 2002), or 12 intersections (Bock & Borisova, 2022), or 14 intersections (Tlauka & Wilson, 1994), or 19 (Waller & Lippa, 2007)

Figure 1 summarizes the outcome of above research. The abscissa indicates the number of intersections along the route, which is a measure of memory load: more intersections require travelers to memorize more directions and/or more cue–direction associations. The abscissa also differentiates between studies: Each study used a fixed number of intersections, and that number was different in all studies. Unfortunately, studies also used different maze topographies, different visual cues, and—most importantly—different outcome measures, such that participants’ performance scores cannot be compared between studies. The ordinate in Fig. 1 is therefore limited to within-study relative scores, where “good” indicates no significant difference from Task SA, and “poorer” indicates a significant decrement compared with Task SA. By definition, performance on Task SA is always “good.” Black bars represent a group of participants examined with Task SA, grey bars represent a different group examined with Task S, and the light-grey bar represents a group examined with Task A. Figure 1 illustrates that performance on Task S was good in studies using six to 14 intersections, but was poorer in a study using 19 intersections. These findings are compatible with the view that the serial order strategy is fully adequate for a moderate number of intersections but is no longer sufficient if that number exceeds the limits of working memory capacity (Hamburger, 2020). If so, Fig. 1 would indicate that those limits are reached with 14 to 18 intersections. Figure 1 also illustrates that performance on Task A was poorer when the route had 12 intersections; unfortunately, no data are available for Task A with another number of intersections.

One possible interpretation for the findings in Fig. 1 is that Task SA with 12 intersections was accomplished by the serial order strategy rather than by the associative cue strategy, since with 12 intersections, performance on Task S was good while performance on Task A was poorer; conversely, Task SA with 19 intersections was possibly accomplished by the associative cue strategy rather than with the serial order strategy, since with 19 intersections, performance on Task S was poorer while performance on Task A might conceivably be good (no data available, though). This interpretation stipulates that in Task SA, the better of the two available strategies is selected and the poorer one is discarded; we will call this the *winner-prevails hypothesis*. This hypothesis implies that the serial order strategy is superior to the associative cue strategy for routes with a moderate number of intersections, but the opposite is the case for routes with a high number of intersections.

An alternative interpretation of Fig. 1 is that Task SA was accomplished by only one strategy, but information that is not essential for that strategy was nevertheless used to enhance performance. For example, Task SA with 19 intersections cannot be accomplished by the serial strategy alone because of the high memory load (cf. Task S with 19 intersections), but it possibly can be accomplished if the serial order strategy is enhanced by exploiting the available visual cues. It has indeed been proposed in the past that visual cues could serve to subdivide the sequence of directions into shorter segments, each of which fits within the limits of working memory capacity (Hamburger, 2020). Thus, a traveler might recall that “left, straight on, straight on” will get her/him from the hotel to the drugstore, “right, straight on, right” from the drugstore to the town hall. Such segmentation is akin to the mnemonic technique of “chunking” (Miller, 1956), with the peculiarity that the chunks are demarcated by spatial cues. As another example, Task SA with 18 intersections might not be accomplishable by the associative cue strategy alone, but it possibly can be accomplished if the associative cue strategy is enhanced by serial order knowledge (e.g., by grouping visual cues encountered at neighboring intersections). We will refer to this interpretation as the *strategy-enhancement hypothesis*.

A third interpretation of Fig. 1 is provided by the *dual*-*strategy hypothesis*. It stipulates that on Task SA with 19 intersections, the memory load is too high for either strategy alone, and the task therefore is accomplished by combining both. Thus, travelers memorize the serial order of directions as well as the cue–direction associations, which constitutes a redundant, dual encoding of directions. It is known that dual encoding of items through independent channels (e.g., spatial and verbal) facilitates subsequent recall of those items (Paivio & Csapo, 1973), and dual encoding of directions might likewise facilitate their recall.

The purpose of our study was to scrutinize the validity of above three hypotheses. To this end, we implemented Tasks S, A, and SA with 12 and with 18 intersections, and we registered participants’ route-following performance as well as their acquired knowledge about serial order and about cue–direction associations. The *winner-prevails hypothesis* makes two alternative predictions about the outcome. According to one, route-following performance will be similar on Tasks SA and S, serial order knowledge after Task SA will be good, but cue–direction knowledge after Task SA will be poor. According to the other alternative, route-following performance will be similar on Tasks SA and A, cue–direction knowledge after Task SA will be good, but serial order knowledge after Task SA will be poor. The *strategy-enhancement hypothesis* also makes two alternative predictions. Route-following performance will be better on Task SA than on Tasks S and A, either serial order knowledge *or* cue–direction knowledge after Task SA will be good, and the other type of knowledge will be poor. The *dual-strategy hypothesis* predicts that route-following performance will be better on Task SA than on Tasks S and A, and serial order knowledge *as well as* cue–direction knowledge will be good. Thus, each of the three hypotheses predicts a different pattern of findings, allowing us to scrutinize the fit between hypotheses and experimental data.

The outcome of this research is of broader theoretical interest, as it evaluates how multiple cognitive processes—here: decision strategies—are coordinated to achieve a desirable behavioral goal—here: to reach a destination. The outcome also is of practical interest, as it is relevant for the design of wayfinding training. As an example, the winner-prevails hypothesis stipulates that training should target either the acquisition of serial order knowledge or the acquisition of cue–direction knowledge, while the other two hypotheses stipulate that training should target the acquisition of both types of knowledge in their natural combination.

The present work implements an established experimental approach which evaluates the decision-making component of wayfinding, and not the locomotion component. In this approach (Bock & Borisova, 2022; Cohen & Schuepfer, 1980; Wiener et al., 2012), seated participants view the still image of an intersection, decide which way to go, then view the still image of the next intersection, decide again, and so on.

## Methods

### Participants

A preliminary sample yielded the effect size *f* = 0.37 for the main effect of interest, which is the effect of “task” on the proportion of correct responses (see below). With *f* = 0.37, α = 0.05, and ß = 0.95, G*Power (Faul et al., 2007) yielded for the main effect of task in the planned four-way analysis of variance (ANOVA; see below) a required sample size of *n* = 72. We decided to test 120 participants. No participant was dropped from the analyses. A second effect of interest is the effect of “preceding task” on the serial order test and the cue association test (see below). With *f* = 0.25, α = 0.05, and ß = 0.95, G*Power (Faul et al., 2007) yielded for the main effect of “preceding task” in the planned two-way ANOVA (see below) a required sample size of *n* = 210. Nonsignificance of “preceding task” should therefore be interpreted with caution, as it may reflect a Type II error.

One hundred and twenty healthy young adults were recruited by word of mouth and by written postings. They were assigned by chance to six experimental groups, whose demographic characteristics are summarized in Table 1. All participants signed an informed consent statement before testing began. The experimental protocol was approved by the Ethics Committee of the German Sport University.

**Table 1:** Characteristics of participants

Group	S12	S18	A12	A18	SA12	SA18
Sample size	20	20	20	20	20	20
Age (*M* ± *SD*)	22 ± 2	25 ± 3	21 ± 2	22 ± 2	22 ± 2	24 ± 2
Females	5	4	10	10	7	4
Education (years)	13 ± 0	15 ± 3	13 ± 1	14 ± 2	13 ± 1	13 ± 1

### Route-following—General procedures

**Fig. 2:**
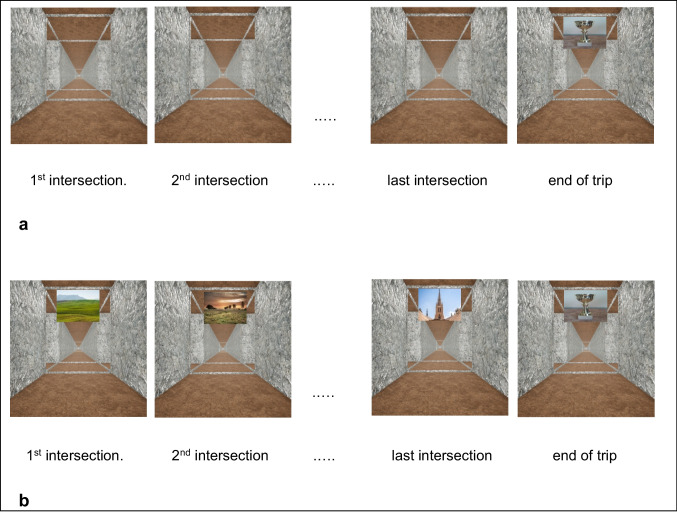
Images presented to (**a**) group S12 and S18 and to (**b**) all other groups

Participants were seated in front of a 15-inch computer monitor at a convenient viewing distance of about 50 cm. If they wore eyeglasses in everyday life, they were asked to wear them during the tests as well. The monitor displayed the color image of a four-way intersection viewed from a first-person perspective (for examples, see Fig. 2). The intersection was created by Unreal Engine® 4.16.2 (Epic Games), a software for the design of virtual environments. The size of the displayed image increased by 50% within the first second of presentation, to increase task realism by a simulated final approach to the intersection.

Participants took six trips along a prescribed route through a virtual maze (for detailed instructions, see the Appendix). The first trip was experimenter-guided. Upon display of an intersection, the experimenter informed participants verbally in which direction the route continued (left, right, or straight on) and then initiated the display of the next intersection, and so on. At the end of the route, a shining trophy was displayed as an immaterial reward (see Fig. 2, rightmost images). The remaining five trips were self-guided. Upon display of an intersection, participants indicated verbally in which direction the route continued. The experimenter confirmed this response verbally, or told the participants what the correct direction was, and then initiated the display of the next intersection. At each intersection, therefore, participants could commit zero or one error, and they did not have to correct their error. Participants were instructed to respond on the self-guided trips at an unhurried pace, whenever they felt ready, which resulted in a stimulus-to-response interval of about 2 to 5 seconds. Performance was quantified as:

1 $$proportion\ of\ correct\ responses=\frac{number\ of\ intersections- number\ of\ errors}{number\ of\ intersections}.$$

Thus, perfect performance would yield a score of 1.00, while random performance would yield a score of 0.33 since there were three alternatives per intersection.

### Route-following—Groups

For group S12 and S18, all intersections looked exactly the same (cf. Fig. 2a). To respond correctly on self-guided trips, therefore, participants had to recall the sequence of directions encountered during the first trip. Group S12 was given a route with twelve intersections, and group S18 was given a route with eighteen intersections.

For group SA12 and SA18, all intersections looked exactly the same except for an intersection-specific cue. This cue hung from the ceiling and depicted a landscape or a building (cf. Fig. 2b). The serial order of cues and of directions was the same on all trips. To respond correctly on self-guided trips, therefore, participants could recall the sequence of directions encountered during the first trip, or they could recall the cue–direction associations encountered during the first trip. Group SA12 was given a route with 12 intersections, and group SA18 was given a route with 18 intersections.

Group A12 and A18 differed from SA12 and SA18 only in that the serial order of cues differed between trips. However, each cue remained associated with the same direction on all trips. For example, a river was associated with a rightward turn on all trips; it was presented at the fifth intersection of the first trip, at the ninth intersection of the second trip, and so forth, and each time it called for a rightward turn. To respond correctly on self-guided trips, therefore, participants had to recall the cue–direction associations encountered during the first trip, but *not* the sequence of directions encountered during the first trip. Group A12 was given a route with 12 intersections, and group A18 was given a route with 18 intersections.

### Other assessments

After signing an informed consent statement and a data privacy statement, participants completed a demographics questionnaire and a modified version of the general self-efficacy scale ASKU (Beierlein et al., 2012). We modified ASKU by replacing references to self-efficacy in general with references to self-efficacy for spatial orientation. Specifically, the questionnaire items were: (1) In a difficult situation, I can rely on my own sense of orientation. (2) In unfamiliar places, I can find the destination by myself. (3) Under complicated conditions, I still can find the right path to go. Response options ranged from 1 = *not true at all* to 5 = *perfectly true*, and the total score, which is the sum of all item scores, therefore could range from 3 to 15.

After completing the questionnaires and one of the route-following tasks, group A12 and A18 were given the *cue association test:* The spatial cues from the route-following task were presented concurrently on a computer monitor, and participants had to name the associated directions. Group SA12 and SA18 were also given this test, and additionally the *serial order test*, which was identical to a trip of the S groups, except that no accuracy feedback was provided. Groups S12 and S18 were not given the cue association test since they had experienced no spatial cues during the route-following task, and they were not given the serial order test since their performance on the last route-following trip served as an equivalent for that test. Total session duration was about 30 minutes for all groups.

### Data analysis

The proportion of correct responses on the route-following task served as a dependent variable for an ANOVA, with the between-factors task (S, A, SA), number of intersections (12, 18), and sex (f, m), and with the within-factor trial (2 to 6). The degrees of freedom were Greenhouse–Geisser adjusted for nonsphericity. We included the factor sex only to control for potential differences between males and females (see, e.g., Boone et al., 2018); our study was not designed to verify the existence of such differences.

Participants’ self-efficacy for spatial orientation was compared with their route-following performance by calculating the Pearson correlation between ASKU scores and the proportion of correct responses on the last trip.

To analyze the outcome of the cue association test, we subtracted 0.33 from the proportion of correct responses on this test, such that random performance would now correspond to a score of zero; the outcome served as a dependent variable for an ANOVA with the between-factors preceding task (A, SA) and ‘number’ (12, 18). Preceding task refers to the route-following task performed prior to the cue association test, and number refers to the number of intersections on the preceding task. The outcome of the serial order test was analyzed in the same way, except that the factor levels of preceding task now were S and SA. Adding the terms sex and sex × preceding task yielded additional statistical significance and were therefore retained to ensure that group differences are not confounded with sex differences. However, we did not interpret any sex-related effect since our sample size is not adequate for this.

## Results

**Fig. 3:**
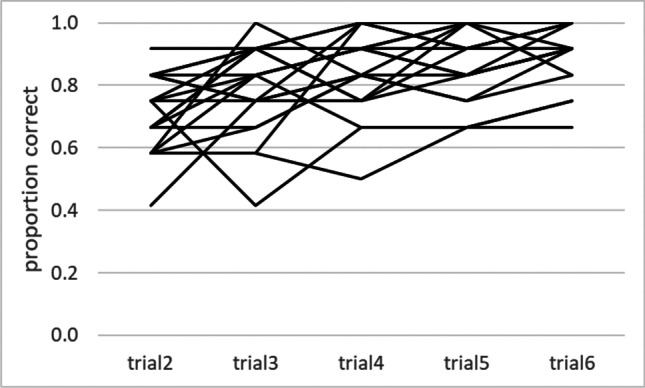
Raw data from group S12 on all self-guided trips. *Note.* Each line represents one participant, and each point along a line represents the proportion of correct responses on a given trip. Random performance would yield a score of 0.33

**Fig. 4:**
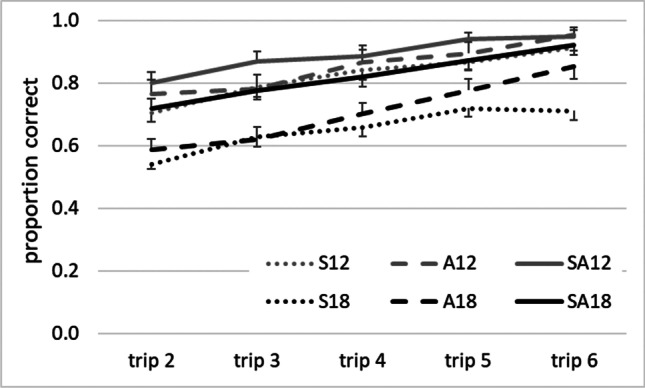
Data from all six groups on all self-guided trips. Each line represents the mean proportion of correct responses from all participants of a given group. Grey and black lines represent groups with 12 and with 18 intersections, respectively. Dotted, dashed and solid lines represent groups on Task S, A, and SA, respectively. To reduce cluttering, error bars depict standard errors rather than standard deviations

Figure 3 depicts exemplary raw data from group S12, to illustrate response variability. Figure 4 summarizes graphically the results from each group, and Table 2 presents the pertinent ANOVA findings. ANOVA yielded a highly significant effect for trip: Performance improved consistently from one self-guided trip to the next. An ANOVA also yielded high significance for number: performance was better with 12 than with 18 intersections. Finally, high significance was also yielded for task. Post hoc decomposition of the latter effect by Tukey’s HSD tests revealed no significant difference between Tasks S and A (*p* = 0.228), but significant differences between S and SA (*p* <0.001) as well as between A and SA (*p* = 0.014): Performance on Task SA was reliably better than that on Tasks S and A. The effect of trip × task just reached significance with a very small effect size: The trial-to-trial increase of performance was somewhat larger on Task A than on Tasks S and SA.

**Table 2:** ANOVA results for route-following

	*df*1	*df*2	*F*	*p*	partial η^2^
constant	1	108	4387.06	<0.001	0.98
task	2	108	8.27	<0.001	0.13
number	1	108	24.95	<0.001	0.19
sex	1	108	1.35	0.248	0.01
task × number	2	108	2.03	0.136	0.04
task × sex	2	108	0.24	0.788	<0.01
number × sex	1	108	1.04	0.309	<0.01
task × number × sex	2	108	0.19	0.830	<0.01
trip	3.41	368.47	77.00	<0.001	0.42
trip × task	6.82	368.47	1.97	0.049	0.04
trip × number	3.41	368.47	0.77	0.543	<0.01
trip× sex	3.41	368.47	2.19	0.069	0.02
trip × task × number	6.82	368.47	0.44	0.899	<0.01
trip × task × sex	6.82	368.47	0.72	0.677	0.01
trip × number × sex	3.41	368.47	0.63	0.644	<0.01
t × t × n × s	6.82	368.47	0.98	0.452	0.02

The correlation between ASKU scores and performance on trip 6 was 0.02 when participants from all groups were considered together, and it ranged between −0.16 and +0.09 when each group was considered separately. None of those correlations were statistically significant (all *p*s > 0.05).

The ANOVA results for the serial order test and for the cue association test are presented in Tables 3 and 4, respectively. Both tests yielded a highly significant constant term, indicating that test performance was reliably better than random. Both tests yielded no significance for preceding task, although serial order knowledge as well as cue–direction knowledge were slightly lower after Task SA then after Task S or A, respectively. The serial order test but not the cue association test also yielded a highly significant effect of number, as it was substantially easier to remember a sequence of 12 rather than 18 directions.

**Table 3:** ANOVA results for the serial order test

	*df*1	*df*2	*F*	*p*	partial η^2^
constant	1	74	2449.02	<0.001	0.97
preceding task	1	74	3.67	0.059	0.05
number	1	74	73.16	<0.001	0.50
sex	1	74	4.42	0.039	0.06
prec. task × number	1	74	0.74	0.391	0.01
prec. task × sex	1	74	9.77	0.003	0.12

For a definition of preceding task and number, see the Methods section

**Table 4:** ANOVA results for the cue association test

	*df*1	*df*2	*F*	*p*	partial η^2^
constant	1	74	1649.79	<0.001	0.96
preceding task	1	74	2.34	0.130	0.03
number	1	74	0.76	0.387	0.01
sex	1	74	5.53	0.021	0.07
prec. task ×number	1	74	1.07	0.303	0.01
prec. task × sex	1	74	3.71	0.058	0.05

For a definition of preceding task and number, see the Methods section

**Fig. 5:**
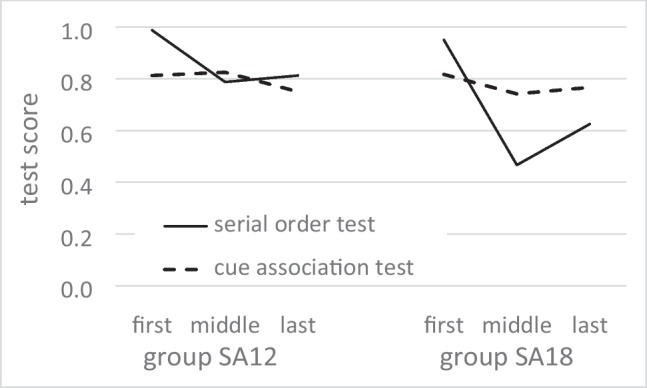
Serial order test and cue association test for route sections. Test scores are plotted separately for the first, middle and last section of the route. Data from group SA12 are shown on the left, and those from group SA18 on the right. Bold lines represent the across-participant means on the serial order test and thin lines those on the cue association test

We conducted two exploratory analyses about the knowledge acquired by group SA12 and SA18. We reasoned that if both strategies are available, there might be trade-off such that the more knowledge of one type is acquired, the less knowledge of the other type is acquired. One analysis explored this tradeoff with respect to differences among participants. We found that individual scores on the serial order test and on the cue association test were indeed negatively correlated, but the correlation was small (*r* = −0.25) and nonsignificant (*p* = 0.166). A second analysis explored the trade-off with respect to different sections of the route. We calculated the scores on the serial order test and on the cue association test separately for the first, the middle, and the last third of the route, and found that those partial scores correlated positively rather than negatively between tests (*r* = 0.73). Figure 5 illustrates that partial scores on the serial order test were distinctly higher for the first than for the second and third section of the route in groups SA12 and SA18, and that partial scores on the cue association test exhibited a subtle trend in the same rather than in the opposite direction. These data are in line with a primacy effect for both types of knowledge, but not with a trade-off.

## Discussion

To follow a prescribed route, travelers must decide which way to proceed across each intersection. For this they can use the serial order strategy and/or the associative cue strategy, since both strategies are typically available in real life. Available data (cf. Fig. 1) leave open whether travelers pick the better strategy and disregard the poorer one (the *winner-prevails hypothesis*), use only one of the strategies and enhance it by the available additional information (the *strategy-enhancement hypothesis*), or use both strategies concurrently to benefit from dual encoding (the *dual*-*strategy hypothesis*). The present study scrutinized the validity of these hypotheses by asking participants to follow a prescribed route when only the serial order strategy was available (Task S), only the associative cue strategy was available (Task A), or both strategies were available (Task SA). The memory load was varied by implementing routes with 12 and with 18 intersections.

In all experimental groups, route-following performance increased from trip to trip at a similar rate, and was generally better with 12 than with 18 intersections. Performance was similar on Task S and A, but was lower on both of these tasks than on Task SA. Inferior performance on Task S than Task SA is in accordance with one earlier study (Waller & Lippa, 2007) but seems to be in conflict with another study that found no significant differences between S and SA (Bock & Borisova, 2022; cf. middle cluster of bars in Fig. 1). However, the latter study involved only two rather than three response alternatives per intersection, and it therefore is conceivable that performance on Task S is only poorer than on Task SA when the number of response alternatives is sufficiently high. This would imply that the limits of working memory capacity can be exceeded not only for a high number of intersections, but also for a high number of response alternatives per intersection. Future research should vary the numbers of intersections and of response alternatives independently, to determine the relative contribution of both quantities to the memory load in route-following.

After completing the route-following task, participants’ knowledge about serial order and about cue–direction associations was determined. The serial order knowledge acquired in Task SA was significantly better than chance, and was comparable to Task S, thus confirming earlier findings (Cohen & Schuepfer, 1980). Similarly, the cue–direction knowledge acquired in Task SA was significantly better than chance, and was comparable with Task A. In Task SA, therefore, participants acquired both types of knowledge.

Summing up, route-following performance was better on Task SA than on Tasks S and A, and serial order knowledge as well as cue–direction knowledge after Task SA were substantial. This pattern of findings is in agreement with the predictions of the *dual-strategy hypothesis*, but not with those of the *winner-prevails hypothesis* or the *strategy-enhancement hypothesis*. Notably, our data meet the predictions of the dual-strategy hypothesis not only for routes with 18 intersections but also for routes with 12 intersections, where the memory load probably did not approach the limits of working memory capacity. Perhaps dual encoding is the default procedure in route-following, not a special procedure reserved for challenging situations.

A potential limitation of our study is that participants did not physically walk along the route, but rather saw a sequence of still slides. Proprioceptive, vestibular, locomotor, and optic-flow information about the gradual progress along the route therefore was absent. Although walking and decision-making are distinct processes in a route-following task (e.g., the decision to turn left rather than right at an intersection) is not influenced by the speed of walking towards that intersection, it remains conceivable that those processes nevertheless influence each other. Thus, walking and decision-making might interfere, because they compete for a common pool of computational resources (Wickens, 2002), or they might enhance each other, because they are integrated by a common, familiar context (Godden & Baddeley, 1975). Experimental evidence from spatial exploration (review in Chrastil & Warren, 2012) and route-following tasks (Ruddle et al., 2011) largely support the integration concept, although evidence for the competition concept has also been yielded (Agathos et al., 2020). Another potential limitation is that our participants responded at an unhurried pace (see Methods); our findings may therefore not generalize to those everyday situations where travelers decide under time pressure.

## Data Availability

The datasets generated during and/or analyzed during the current study are available from the corresponding author on reasonable request

## Appendix: Instructions for the route-learning task

### Instructions before the first trip

“In this task, you have to find the correct route through a maze. For that, you have to decide at each intersection, whether the route continues straight on, turns left or turns right. On the first trip, I will tell you at each intersection in which direction you have to go. On the following trips, you will tell me in which direction you want to go, and I will correct you if necessary.”

#### Additionally, for groups SA12 and SA18

“At each intersection, you will see a different picture that will help you to determine the correct direction.”

#### Additionally, for groups A12 and A18

“At each intersection, you will see a different picture that will help you to determine the correct direction. Importantly, the order of intersections will vary from trip to trip, and so will the order of pictures and the order of directions. For example, if you see the picture of a yellow house at the fifth intersection of the first trip and I tell you to turn left there, then you should turn left at the yellow house on all subsequent trips, even if the yellow house appears at the tenth or another intersection.”

### Instructions before the second trip

“Now, you are on your own. Please, tell me at each intersection in which direction you want to go, and I will correct you if necessary. You don’t need to hurry, answer whenever you are ready.”

## Declarations

The corresponding author is familiar with the Psychonomic Society’s Statistical Guidelines.

The manuscript includes no copyrighted material.
